# Supplementary material for: Mechanisms governing the pioneering and redistribution capabilities of the non-classical pioneer PU.1
Source: Nat Commun. 2020 Jan 21;11:402. doi: 10.1038/s41467-019-13960-2 (PMC6972792; doi:10.1038/s41467-019-13960-2)
Supplement: Supplementary file 7 — Source data [file 41467_2019_13960_MOESM7_ESM.zip › Source_Data/FigureS5/FigureS5B_MotifsETS1specificPeaks/homerResults.html]

/misc/data/analysis/project\_PU1/CTV1/motifs/basic/ETS1vsFLI1.DSG\_PU1.peaks.DESEQ.bg/final - Homer de novo Motif Results


# Homer *de novo* Motif Results (/misc/data/analysis/project\_PU1/CTV1/motifs/basic/ETS1vsFLI1.DSG\_PU1.peaks.DESEQ.bg/final)

Known Motif Enrichment Results  
Gene Ontology Enrichment Results  
If Homer is having trouble matching a motif to a known motif, try copy/pasting the matrix file into
STAMP  
More information on motif finding results: HOMER
| Description of Results
| Tips
  
Total target sequences = 1135  
Total background sequences = 8161  
\* - possible false positive  

|  |  |  |  |  |  |  |  |  |
| --- | --- | --- | --- | --- | --- | --- | --- | --- |
| Rank | Motif | P-value | log P-pvalue | % of Targets | % of Background | STD(Bg STD) | Best Match/Details | Motif File |
| 1 | G T C A G T A C A G C T G T C A A G T C T C G A C T G A G A C T A G C T G A T C A G T C G T A C C T G A A C T G | 1e-205 | -4.723e+02 | 31.28% | 2.31% | 44.6bp (78.3bp) | GFY(?)/Promoter/Homer(0.938) More Information \| Similar Motifs Found | motif file (matrix) |
| 2 | T G A C T G C A C T A G C T A G C G T A A C G T A C T G A G C T A C T G T C A G A C G T G A T C C G T A | 1e-179 | -4.129e+02 | 32.07% | 3.42% | 24.3bp (43.9bp) | ETS:RUNX(ETS,Runt)/Jurkat-RUNX1-ChIP-Seq(GSE17954)/Homer(0.937) More Information \| Similar Motifs Found | motif file (matrix) |
| 3 | C T G A C T G A C G T A A G T C A C G T C G T A A G T C C T G A | 1e-126 | -2.915e+02 | 28.28% | 4.32% | 47.6bp (73.4bp) | Rbm42(RRM)/Xenopus\_tropicalis-RNCMPT00282-PBM/HughesRNA(0.881) More Information \| Similar Motifs Found | motif file (matrix) |
| 4 | A G T C C T G A C T G A G A C T A C G T A T G C A G T C A G T C C G T A T A C G | 1e-95 | -2.194e+02 | 27.75% | 5.97% | 50.1bp (67.4bp) | ZNF416(Zf)/HEK293-ZNF416.GFP-ChIP-Seq(GSE58341)/Homer(0.759) More Information \| Similar Motifs Found | motif file (matrix) |
| 5 | T A G C A T G C G C T A C T A G T A C G C G T A A C G T A C T G | 1e-89 | -2.062e+02 | 31.54% | 8.42% | 47.7bp (57.4bp) | SPDEF(ETS)/VCaP-SPDEF-ChIP-Seq(SRA014231)/Homer(0.797) More Information \| Similar Motifs Found | motif file (matrix) |
| 6 | C T A G C T A G C G T A A G T C A C G T C G T A A G T C G T C A C T G A C G A T G A C T T G A C | 1e-69 | -1.597e+02 | 11.63% | 0.94% | 55.2bp (82.7bp) | GFY(?)/Promoter/Homer(0.768) More Information \| Similar Motifs Found | motif file (matrix) |
| 7 | C A G T C A T G G T A C T G C A A G C T A C G T A G T C G C A T C T A G A C T G A T C G C T G A | 1e-67 | -1.543e+02 | 14.01% | 1.76% | 54.8bp (66.6bp) | ZNF143\|STAF(Zf)/CUTLL-ZNF143-ChIP-Seq(GSE29600)/Homer(0.867) More Information \| Similar Motifs Found | motif file (matrix) |
| 8 | T A C G T G C A G T A C G A C T T A G C G T A C G C T A G C T A A G T C G C A T A T G C | 1e-65 | -1.502e+02 | 19.30% | 4.05% | 59.7bp (69.4bp) | Pp\_0237(RRM)/Physcomitrella\_patens-RNCMPT00237-PBM/HughesRNA(0.769) More Information \| Similar Motifs Found | motif file (matrix) |
| 9 | T A G C T A C G C T A G T G C A T A G C A C G T T G C A A T G C | 1e-53 | -1.237e+02 | 32.33% | 12.94% | 54.8bp (74.6bp) | Rbm42(RRM)/Xenopus\_tropicalis-RNCMPT00282-PBM/HughesRNA(0.786) More Information \| Similar Motifs Found | motif file (matrix) |
| 10 | A T G C C T A G C G A T G A T C A G T C A C G T T C A G | 1e-52 | -1.205e+02 | 41.41% | 19.88% | 56.3bp (72.7bp) | ZmHOX2a(1)(HD-HOX)/Zea mays/AthaMap(0.840) More Information \| Similar Motifs Found | motif file (matrix) |
| 11 | A C G T A G T C A T G C T G A C C G T A C T G A T C A G C T G A | 1e-39 | -9.066e+01 | 32.95% | 15.74% | 53.7bp (68.4bp) | Rbpj1(?)/Panc1-Rbpj1-ChIP-Seq(GSE47459)/Homer(0.806) More Information \| Similar Motifs Found | motif file (matrix) |
| 12 | T G A C C G A T T C A G A C T G G T C A A G T C G A C T C T A G G T A C T C G A | 1e-30 | -6.956e+01 | 8.72% | 1.72% | 50.4bp (63.8bp) | ZFX(Zf)/mES-Zfx-ChIP-Seq(GSE11431)/Homer(0.669) More Information \| Similar Motifs Found | motif file (matrix) |
| 13 | C T G A T C A G T G C A T C G A A C G T A T C G A G T C G T A C T A G C G A T C C T A G | 1e-28 | -6.536e+01 | 19.74% | 8.22% | 48.6bp (71.1bp) | ABI4(2)(AP2/EREBP)/Zea mays/AthaMap(0.674) More Information \| Similar Motifs Found | motif file (matrix) |
| 14 | A C T G G A C T C G T A T A C G A G C T A G C T A G T C A G C T T A G C C A T G A C T G C A T G A C T G A G T C | 1e-24 | -5.535e+01 | 4.49% | 0.46% | 50.0bp (61.4bp) | Rbm42(RRM)/Xenopus\_tropicalis-RNCMPT00282-PBM/HughesRNA(0.676) More Information \| Similar Motifs Found | motif file (matrix) |
| 15 | A T C G C G T A A C G T A G C T T A G C C T A G G A C T C G T A A C T G A C G T | 1e-23 | -5.524e+01 | 4.41% | 0.44% | 57.9bp (63.7bp) | Rbm42(RRM)/Xenopus\_tropicalis-RNCMPT00282-PBM/HughesRNA(0.761) More Information \| Similar Motifs Found | motif file (matrix) |
| 16 | T A G C A T C G A G T C C T A G T A G C A C T G A C T G A G T C T C G A A G T C A C T G A T G C | 1e-22 | -5.239e+01 | 11.54% | 3.86% | 45.1bp (83.5bp) | PHO2/PHO2\_H2O2Hi/[](Harbison)/Yeast(0.753) More Information \| Similar Motifs Found | motif file (matrix) |
| 17 | A G T C G T A C A T C G T G C A C A T G C G T A G T C A | 1e-21 | -5.009e+01 | 33.30% | 20.06% | 55.4bp (66.8bp) | PB0138.1\_Irf4\_2/Jaspar(0.860) More Information \| Similar Motifs Found | motif file (matrix) |
| 18 | A G T C A T G C T G C A C T A G T G C A C T A G C T A G G A T C G A T C | 1e-21 | -4.862e+01 | 21.32% | 10.72% | 55.0bp (67.8bp) | PB0189.1\_Tcfap2a\_2/Jaspar(0.742) More Information \| Similar Motifs Found | motif file (matrix) |
| 19 | A C T G A G T C A G T C A C T G A C T G A C T G C G T A | 1e-17 | -4.049e+01 | 20.62% | 10.99% | 53.9bp (77.4bp) | PUT3/MA0358.1/Jaspar(0.793) More Information \| Similar Motifs Found | motif file (matrix) |
| 20 | A T G C A G T C G A T C G A C T G T A C C A G T T A C G C T G A A T C G C G T A T C G A C G T A C A G T | 1e-17 | -4.012e+01 | 4.58% | 0.81% | 50.3bp (53.2bp) | STAT5(Stat)/mCD4+-Stat5-ChIP-Seq(GSE12346)/Homer(0.662) More Information \| Similar Motifs Found | motif file (matrix) |
| 21 | T C A G G C T A C T G A C T G A A C T G C G T A A T C G A G T C G A T C T C A G C G T A T C G A | 1e-16 | -3.701e+01 | 1.76% | 0.04% | 44.8bp (11.0bp) | Deaf1/dmmpmm(Pollard)/fly(0.627) More Information \| Similar Motifs Found | motif file (matrix) |
| 22 | A T C G A C G T A G T C A C T G A T C G A G T C A T G C G A T C A T C G A G T C T C G A A G C T A C G T | 1e-15 | -3.515e+01 | 1.59% | 0.02% | 51.7bp (4.7bp) | SAMD4A(SAM)/Homo\_sapiens-RNCMPT00063-PBM/HughesRNA(0.714) More Information \| Similar Motifs Found | motif file (matrix) |
| 23 | T C G A C T A G A T C G A G T C G C T A T G A C A G T C A T C G A G T C A T C G T A G C G A T C T A G C T A C G | 1e-15 | -3.462e+01 | 3.44% | 0.51% | 49.8bp (84.3bp) | Sp1(Zf)/Promoter/Homer(0.695) More Information \| Similar Motifs Found | motif file (matrix) |
| 24 | A G C T G T C A A C T G G T C A C G T A A C T G T A C G C G T A C G A T A C T G | 1e-14 | -3.411e+01 | 2.91% | 0.33% | 54.1bp (31.0bp) | Tb\_0230(RRM)/Trypanosoma\_brucei-RNCMPT00230-PBM/HughesRNA(0.748) More Information \| Similar Motifs Found | motif file (matrix) |
| 25 | A G C T A C T G A T G C G T A C A G T C A G T C A C T G A T C G A T C G A T C G A T G C A T G C A G C T | 1e-13 | -3.181e+01 | 5.64% | 1.60% | 49.2bp (70.7bp) | TFAP2A(var.2)/MA0810.1/Jaspar(0.743) More Information \| Similar Motifs Found | motif file (matrix) |
| 26 | T G A C G C A T G C A T G A T C A C G T A T G C C G T A T C A G C G T A | 1e-13 | -3.180e+01 | 17.80% | 9.80% | 58.7bp (59.5bp) | STAT5(Stat)/mCD4+-Stat5-ChIP-Seq(GSE12346)/Homer(0.771) More Information \| Similar Motifs Found | motif file (matrix) |
| 27 | C G A T A T G C A C G T A C G T A C G T A T G C A G T C A T G C A C G T A T C G C T G A A C T G | 1e-13 | -3.114e+01 | 1.59% | 0.05% | 58.0bp (42.3bp) | PCBP3(KH)/Mus\_musculus-RNCMPT00215-PBM/HughesRNA(0.775) More Information \| Similar Motifs Found | motif file (matrix) |
| 28 | C A G T C A G T C A T G C G A T A C G T A G T C C G A T G T A C G C A T A C T G | 1e-12 | -2.882e+01 | 8.63% | 3.54% | 52.2bp (55.2bp) | Ot\_0263(RRM)/Ostreococcus\_tauri-RNCMPT00263-PBM/HughesRNA(0.775) More Information \| Similar Motifs Found | motif file (matrix) |
| 29 | C G A T A C T G A T G C A C G T G A T C G C T A G A T C C G T A A C T G G T A C A G T C | 1e-12 | -2.843e+01 | 2.47% | 0.30% | 57.3bp (51.6bp) | Tbx20(T-box)/Heart-Tbx20-ChIP-Seq(GSE29636)/Homer(0.657) More Information \| Similar Motifs Found | motif file (matrix) |
| 30 | T A C G T C G A C T A G T C G A T C G A G C A T T A G C G T A C T A C G G C T A T A C G | 1e-12 | -2.806e+01 | 12.95% | 6.58% | 55.3bp (62.8bp) | ZNF519(Zf)/HEK293-ZNF519.GFP-ChIP-Seq(GSE58341)/Homer(0.708) More Information \| Similar Motifs Found | motif file (matrix) |
